# Using genderize.io to infer the gender of first names: how to improve the accuracy of the inference

**DOI:** 10.5195/jmla.2021.1252

**Published:** 2021-10-01

**Authors:** Paul Sebo

**Affiliations:** 1 paulsebo@hotmail.com, Primary Care Unit, Faculty of Medicine, University of Geneva, Geneva, Switzerland

**Keywords:** accuracy, gender determination, genderize.io, misclassification, name, name-to-gender, performance

## Abstract

**Objective:**

We recently showed that genderize.io is not a sufficiently powerful gender detection tool due to a large number of nonclassifications. In the present study, we aimed to assess whether the accuracy of inference by genderize.io can be improved by manipulating the first names in the database.

**Methods:**

We used a database containing the first names, surnames, and gender of 6,131 physicians practicing in a multicultural country (Switzerland). We uploaded the original CSV file (file #1), the file obtained after removing all diacritic marks, such as accents and cedilla (file #2), and the file obtained after removing all diacritic marks and retaining only the first term of the compound first names (file #3). For each file, we computed three performance metrics: proportion of misclassifications (errorCodedWithoutNA), proportion of nonclassifications (naCoded), and proportion of misclassifications and nonclassifications (errorCoded).

**Results:**

naCoded, which was high for file #1 (16.4%), was reduced after data manipulation (file #2: 11.7%, file #3: 0.4%). As the increase in the number of misclassifications was small, the overall performance of genderize.io (i.e., errorCoded) improved, especially for file #3 (file #1: 17.7%, file #2: 13.0%, and file #3: 2.3%).

**Conclusions:**

A relatively simple manipulation of the data improved the accuracy of gender inference by genderize.io. We recommend using genderize.io only with files that were modified in this way.

## INTRODUCTION

Gender detection tools are increasingly used in medical research, particularly to explore the gender gap in scientific publications, grants allocations, salaries, or career advancement processes [1–3]. Their main advantage lies in the possibility of uploading large CSV or Excel files. After processing the data, a new column (gender) is added to the file. This procedure does not require extensive computer skills.

For example, using Gender API [4], we found that the proportion of female first authors for all studies and reviews published between 2016 and 2020 in the sixteen highest-impact primary health care journals was 54%, but this proportion was only 41% for those published in the sixteen highest-impact general internal medicine journals [1]. Using genderize.io [5], Cevik et al. found that women were significantly underrepresented as principal investigators of COVID-19 studies (proportion of female researchers: 28%) [2]. Also using genderize.io, Gottlieb et al. found that only 16% of editorial board members of emergency medicine journals were women [3].

These examples show that gender detection tools can be useful to researchers by saving time and resources. However, determining the gender of individuals based on their first name is not an easy task and raises important ethical issues by oversimplifying the concept of gender [6, 7]. In particular, the concepts of sex (determining the biological aspects of individuals) and gender (essentially a social and cultural construct) are not interchangeable. Also, the dichotomization of gender into feminine or masculine risks marginalizing some individuals who do not recognize themselves in this binary differentiation. Determining gender through self-identification would be preferable and would also increase the accuracy of the data collected. However, self-identification is resource intensive and often not feasible for large-scale studies.

We recently showed that Gender API [4] and NamSor [8] are the most powerful tools for determining the gender of individuals [9]. By contrast, genderize.io [5] does not perform well due to a large number of unclassified first names. However, genderize.io offers researchers a significant advantage over the other two gender detection tools in that it allows researchers to upload a file of 1,000 first names every day for free (i.e., to perform 30,000 queries per month), whereas Gender API is only free up to 500 requests per month and NamSor up to 5,000 requests per month. One way to improve the quality of inference by genderize.io is to use a second gender detection tool for unrecognized first names [9]. Although potentially more accurate, this strategy is relatively time consuming, as it requires creating a new file of these first names and then submitting it to the second gender detection tool.

Therefore, in the present study, our objective was to assess whether the accuracy of inference by genderize.io can be improved by manipulating the first names in the file.

## METHODS

For this study, we used the same database of physicians that we used in our earlier study [9]. This database consisted of 6,264 physicians, 50.4% of whom were women. More specifically, it included 2,183 physicians and 908 trainee physicians practicing at the University Hospital of Geneva (the largest hospital in Switzerland), 207 senior physicians practicing in Swiss university hospitals, 510 community-based physicians practicing in the canton of Geneva, and 2,456 community-based primary care physicians, pediatricians, and gynecologists practicing in Switzerland. The database was built in January 2020. After removing duplicates, it contained the first name, surname, and gender of 6,131 physicians. Gender was known for all physicians in the database and was determined by self-identification.

According to nationalize.io, a tool that predicts the nationality of individuals based on their first name, the most common origins of the first names in the database were French-speaking (32.2%) and English-speaking (14.4%) countries (Appendix 1). The majority of the first names (88.4%) were from Western countries or countries whose main language is one that is spoken in Western countries. The tool failed to assign a country of origin to 916 names (14.9%).

When uploading the original database as a CSV file (file #1), we found that first names with diacritical marks, such as accents and cedilla, and compound first names with or without hyphens were often not recognized by genderize.io. We therefore created two additional files: one with all diacritical marks removed (file #2) and one with all diacritical marks removed keeping only the first term of the compound first names (file #3). We used STATA version 15.1 (College Station, TX, USA) to remove all diacritical marks (with the ustrto and ustrnormalize commands) and shorten all compound names (with the trim, substr, and strpos commands).

For each file, we computed three performance metrics: (1) errorCodedWithoutNA, which is the proportion of misclassifications (i.e., wrong gender assigned to a first name) excluding nonclassifications (i.e., no gender assigned); (2) naCoded, which is the proportion of nonclassifications; and (3) errorCoded, which is the proportion of misclassifications and nonclassifications [10].

## RESULTS

Table 1 shows the confusion matrix for the three datasets: the original file (file #1) and the two manipulated files (files #2 and #3). Table 2 shows the performance metrics for the same three datasets. As shown in Table 1, the high number of nonclassifications for file #1 (n=1,007) decreased substantially after manipulation, especially for file #3 (n=27). In contrast, the number of misclassifications increased only slightly (file #1: 76, file #2: 84, file #3: 112). These results were confirmed by the performance metrics (Table 2). Using errorCoded, which penalizes both types of error equally, we obtained the following results: file #1: 17.7%, file #2: 13.0%, and file #3: 2.3%.

**Table 1:** Confusion matrices for genderize.io (n=6,131 physicians)

Genderize.io	Classified as women n (%)	Classified as men n (%)	Nonclassified n (%)
Original database (file #1)			
Women	2,519 (81.7)	59 (1.9)	507 (16.4)
Men	17 (0.6)	2,529 (83.0)	500 (16.4)
Database without diacritic marks for first names (file #2)			
Women	2,663 (86.3)	66 (2.2)	356 (11.5)
Men	18 (0.6)	2,670 (87.7)	358 (11.7)
Database without diacritic marks for first names and with only the first term for compound first names (file #3)			
Women	2,987 (96.8)	86 (2.8)	12 (0.4)
Men	26 (0.8)	3,005 (98.7)	15 (0.5)

**Table 2:** Performance metrics for genderize.io (n=6,131 physicians)

Genderize.io	errorCoded^*^	errorCodedWithoutNA^†^	naCoded^‡^
Original database (file #1)	0.1766	0.0148	0.1643
Database without diacritic marks for first names (file #2)	0.1302	0.0155	0.1165
Database without diacritic marks for first names and with only the first term for compound first names (file #3)	0.0227	0.0184	0.0044

* errorCoded = the proportion of misclassifications (i.e., wrong gender assigned to a first name) and nonclassifications (i.e., no gender assigned)

† errorCodedWithoutNA = the proportion of misclassifications excluding nonclassifications

‡ naCoded = the proportion of nonclassifications

## DISCUSSION

By removing all diacritical marks and shortening all compound first names, we were able to greatly improve the accuracy of gender inference by genderize.io. As the proportion of unclassified first names decreased substantially while the proportion of misclassified first names increased only slightly, the overall performance of the tool (i.e., errorCoded) improved from 17.7% for file #1 to 2.3% for file #3.

The increase in misclassification can be explained by the loss of information associated with the simplification of first names. For example, shortening the French first name Jean-Pierre (which is only masculine) yields Jean, which is both a feminine English first name and a masculine French first name. Interestingly, the thirty-six additional misclassifications between file #1 and file #3 were more than offset by the substantial increase in the number of correct classifications (+944). We recommend using genderize.io only with files that were modified in this way, as the proportion of nonclassifications was very high in file #1 (naCoded 16.4%). By comparing the results obtained with this double manipulation of first names with those already published in our earlier study [9], we observe that genderize.io is almost as efficient as Gender API (errorCoded 1.8%) and NamSor (errorCoded 2.0%), the two gender detection tools that were shown to be the most powerful.

Our study has two main limitations. First, it was conducted with a database of physicians practicing only in Switzerland. However, this is a multicultural and multilingual country, and nationalize.io showed multiple origins of the first names, even though almost half (i.e. 47%) were of French- or English-speaking origin [9]. Although the results of this study may be generalizable to most Western names, with other names, for example Asian or Middle Eastern, the effectiveness of the method used in the study is yet to be demonstrated. Second, as previously mentioned, the dichotomization of individuals as women or men oversimplifies the concept of gender and raises important ethical issues [6, 7].

In conclusion, we showed that the use of genderize.io led to a substantial number of nonclassifications, as first names with diacritical marks, such as accents and cedillas, and compound first names with or without hyphens were often not recognized by the tool. We also showed that with a relatively simple manipulation of the first names in the database, which can be done either manually or automatically with specific commands (e.g., in Stata), we could substantially increase the performance of the tool. Therefore, we recommend the use of either genderize.io with prior data manipulation or another gender detection tool. Further studies would be useful to assess whether the procedure used in this study also leads to accurate results with non-Western names.

## Data Availability

Data associated with this article are available in the Open Science Framework (https://osf.io/kr2mx/).

## SUPPLEMENTAL FILES

**Appendix 1.** Origin of physicians' first names (n=6,131 physicians)
