# Supplementary material for: Using genderize.io to infer the gender of first names: how to improve the accuracy of the inference
Source: J Med Libr Assoc. 2021 Oct 1;109(4):609–12. doi: 10.5195/jmla.2021.1252 (PMC8608220; doi:10.5195/jmla.2021.1252)
Supplement: Supplementary file 1 — Appendix 1. Origin of physicians' first names (n=6,131 physicians) [file jmla-109-4-609-s01.docx]

Appendix 1. Origin of physicians’ first names (n=6,131 physicians)

| Origin | n^1^ (%) |
| --- | --- |
| French-speaking country | 1679 (32.2) |
| English-speaking country | 751 (14.4) |
| Spanish-speaking country | 404 (7.7) |
| Asian country^2^ | 344 (6.6) |
| Eastern European country | 324 (6.2) |
| Italian-speaking country | 288 (5.5) |
| Western European country^2^ | 272 (5.2) |
| Arabic-speaking country | 259 (5.0) |
| German-speaking country | 259 (5.0) |
| Northern European country^2^ | 220 (4.2) |
| Southern European country^2^ | 217 (4.2) |
| Portuguese-speaking country | 198 (3.8) |

^1^ The total number of physicians does not add to 6,131 because of missing values (no assignments for 916 physicians (14.9%))

^2^ If not already classified in another group (e.g., the Arabic-speaking country group for some Asian countries)
